# Expanding the potential of monoclonal antibodies against interleukin-4 and interleukin-13 in genodermatoses: A case series on the efficacy and safety of dupilumab in epidermolysis bullosa and ichthyosis

**DOI:** 10.1016/j.jdcr.2025.02.032

**Published:** 2025-03-13

**Authors:** Francesca Caroppo, Roberto Mazzetto, Elisa Milan, Fortunato Cassalia, Anna Belloni Fortina

**Affiliations:** a Dermatology Unit, Department of Medicine (DIMED), University of Padua, Padua, Italy; b Pediatric Dermatology Regional Center, Department of Women and Children's Health (SDB) University of Padua, Padua, Italy; c European Network for Rare Skin Disorders (ERN-Skin), Padua, Italy

**Keywords:** dupilumab, epidermolysis bullosa, genodermatoses, ichthyosis, itch, pain, quality of life, rare skin diseases

## Introduction

Genodermatoses are a group of rare genetic diseases characterized by alterations in skin keratinization, which can significantly affect patients' quality of life.

Recent studies have shown an upregulation of various Th-1 and Th-2 cytokines in patients with certain genodermatoses, including ichthyosis and epidermolysis bullosa (EB). Additionally, emerging evidence suggests the potential effectiveness of biological therapies in treating these conditions.^1^

In this case series, we present our clinical experience with the use of dupilumab in 7 patients (3 adults and 4 children) diagnosed with ichthyoses and EB, shedding light on its potential benefits in this challenging therapeutic context.

## Case series

Data were collected from 7 patients referred to the Paediatric and Rare Diseases Regional Center at the University of Padova between January 2021 and September 2023. The cohort included 5 males (71.4%) and 2 females (28.6%), with a mean age of 18.29 years (±11.9).

Questionnaires were administered at baseline and every 3 months during the first year of therapy. After completing the first year, reassessments were conducted every 6 months.

Internationally validated questionnaires were utilized to evaluate quality of life. For patients older than 16 years, the Dermatology Life Quality Index (DLQI)^2^ was applied, while an adapted and validated version, the Children’s DLQI,^3^ was used for those younger than 16 years.

The numerical rating scale (NRS), modified according to Wong-Baker's version^4^ for patients younger than 16 years of age, was used to assess pain and itching.

Epidemiological data and disease distribution for each patient are summarized in Table I.

**Table I:** Clinical characteristics, disease details, genetic mutations, and reported adverse effects in patients affected by severe genodermatoses treated with dupilumab

*N*	Age	Sex	Disease	Mutation	Inheritance	Drug start	Adverse effects
1	45	F	DEB	COL7A1	Autosomic recessive	Sep-21	/
2	10	F	DEB	COL7A1	Autosomic recessive	Feb-22	/
3	14	M	DEB	COL7A1	Autosomic recessive	Mar-22	/
4	19	M	EBS	KRT14	Autosomic recessive	May-22	Eye discomfort
5	11	M	Ichthyosis	NIPAL4	Autosomic recessive	Jun-22	/
6	22	M	Ichthyosis	N/A	N/A	Sep-21	/
7	7	M	Ichthyosis	ABCA12	Autosomic recessive	Feb-23	/

*ABCA12*, ATP-binding cassette subfamily A member 12; *COL7A1*, collagen type VII alpha 1 chain; *DEB*, dystrophic epidermolysis bullosa; *KRT14*, keratin 14; *NIPAL4*, NIPA-like domain containing 4.

**Fig 1:**
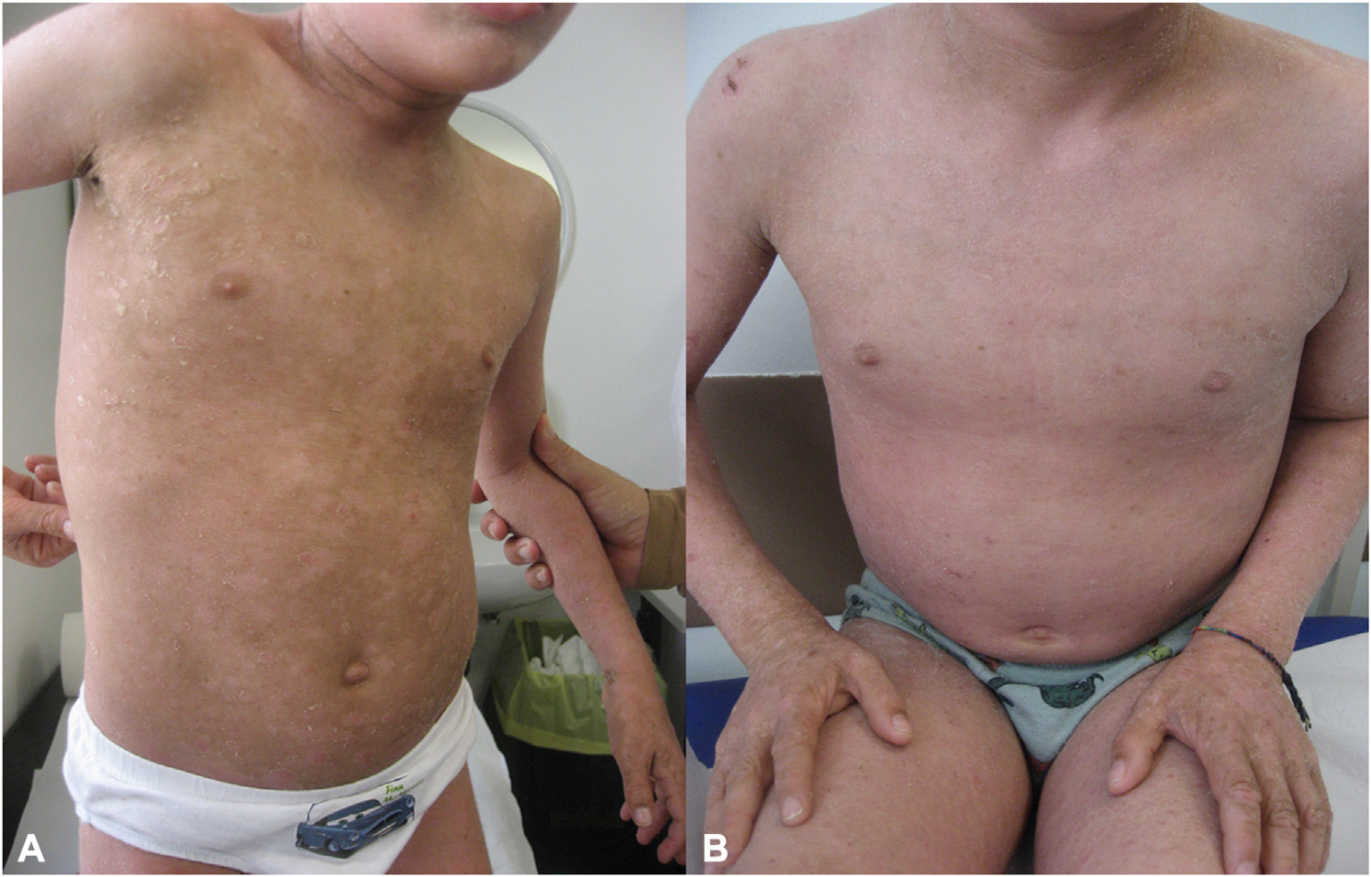
Eleven-year-old boy (patient number 5) affected by ichthyosis and autism. Anterior trunk at baseline (**A**) and after 6 months of therapy with dupilumab (**B**).

Patient 5, an 11-year-old male with congenital ichthyosis and severe autism, was unable to complete the questionnaires. However, his clinical outcomes are presented in Fig 1, *A* and *B*.

The drug dosage followed the recommended guidelines for treating atopic dermatitis, adjusted according to the patient’s age and weight. Questionnaires results assessing quality of life are summarized in Table II.

**Table II:** Schematic summary of the DLQI, NRS pain, and NRS pruritus questionnaires, including changes from baseline, in subjects with epidermolysis bullosa and ichthyosis

	Baseline	3m	%3m/BL	6m	%6m/BL	9m	%9m/BL	12m	%12m/BL
DLQI									
Epidermolysis bullosa patients									
1	10	8	20	7	30	7	30	7	30
2	14	8	42.9	6	57.1	4	71.4	4	71.4
3	28	26	7.1	25	10.7	25	10.7	25	10.7
4	12	10	16.7	8	33.3	8	33.3	5	58.33
Mean	16.00	13.00	21.68	11.50	32.78	11.00	36.35	10.25	42.61
Ichthyosis patients									
5	/	/	/	/	/	/	/	/	/
6	23	2	91.3	2	91.3	2	91.3	2	91.3
7	5	5	0	3	40	/	/	/	/
Mean	14.00		45.65		65.65		91.3		91.3
NRS pain									
Epidermolysis bullosa patients									
1	6	5	16.7	4	33.3	4	33.3	4	33.3
2	8	8	0	6	25	6	25	2	75
3	9	8	11.1	7	22.2	7	22.2	7	22.2
4	8	7	12.5	5	37.5	4	50	3	62.5
Mean	7.75	7.00	10.08	5.50	29.50	5.25	32.63	4.00	48.25
Ichthyosis patients									
5	/	/	/	/	/	/	/	/	/
6	8	2	75	1	87.5	1	87.5	1	87.5
7	6	5	16.7	5	16.7	/	/	/	/
Mean	7.00	3.50	45.85	3.00	52.10	1.00	87.5	1.00	87.5
NRS itch									
Epidermolysis bullosa patients									
1	8	5	37.5	4	50	4	50	4	50
2	8	7	12.5	6	25	5	37.5	4	50
3	8	6	25	6	25	5	37.5	5	37.5
4	10	9	10	6	40	4	60	2	80
Mean	8.50	6.75	21.25	5.50	35.00	4.50	46.25	3.75	54.38
Ichthyosis patients									
5	/	/	/	/	/	/	/	/	/
6	10	2	80	2	80	2	80	2	80
7	7	6	14.3	5	28.6	/	/	/	/
Mean	8.50	4.00	47.15	3.50	54.30	2.00	80.00	2.00	80.00

%Xm/BL = percentage change at month X from baseline.

*DLQI*, Dermatology Life Quality Index; *NRS*, numerical rating scale.

## Discussion

### Epidermolysis bullosa

In our study, 3 patients were diagnosed with dystrophic epidermolysis bullosa (DEB) with mutations in the collagen VII alpha-1 polypeptide gene and 1 patient had an epidermolysis bullosa simplex (EBS), Dowling-Meara type, with a mutation in the keratin 14 gene.

The clinical rationale for using dupilumab to treat these severe forms of EB is based on recent scientific discoveries about the activation of a complex cytokine cascade, which contributes to itching and pain.^5^ Dupilumab can limit this cascade effectively by inhibiting the signaling pathways of both interleukin-4 and interleukin-13, playing a critical role in modulating Th2-mediated inflammation. This makes it a promising therapeutic option for alleviating symptoms in patients with EB.

In the study by Wu PC et al, 5 cases reported halving of the visual analog scale scores for itch after 3 months of therapy.^5^

Moreover, Roque Quintana et al documented the efficacy of dupilumab, with a reduction in DLQI scores from 13 (baseline) to 5 after 9 months of therapy.^6^

The clinical improvements observed in our study are consistent with these findings, although our results suggest a slower response to therapy. In our sample, the maximum reduction in DLQI (42.6%) was achieved after 12 months of therapy, compared to the 3-9 months reported in other studies.^5^^,^^6^

A similar delayed response was observed in itch scores. While studies have reported a halving of itch at 3-5 months, our cohort achieved a comparable reduction (42.6% from baseline) only after 12 months.^5^

These differences may be attributed to the higher baseline itch NRS scores in our patients, which likely required more time to reach significant improvement compared to other studies.^5^^,^^6^

Interestingly, the article by Martora et al reported an ineffectiveness of dupilumab in treating EB, suggesting that the poor response might be due to a distinct cytokine profile in EBS compared to EBD.^7^

However, in our cohort, the patient with EBS (case 4 in Table I) demonstrated significant improvement, achieving a 58.3% reduction in DLQI scores after 12 months of treatment.

A comparative analysis of our findings with existing literature on the efficacy and safety of dupilumab in patients with DEB is summarized in Table III.

**Table III:** Comparative analysis of our study findings with existing literature on the efficacy and safety of dupilumab in patients with ichthyosis, dystrophic epidermolysis bullosa (DEB), and epidermolysis bullosa simplex (EBS)

Authors (year)	Dermatological studied condition	Number of patients	Duration of treatment	Age	Results
Epidermolysis bullosa					
Wu PC et al (2023) systematic review^5^	DEB COL7A1	5	1-3 mo	10-35	**VAS halved** after 3 mo of therapy. **A reduction in DLQI from 23 to 8 and from 13 to 2.5** was reported after 3 mo of therapy
Wu XG et al (2023)^8^	DEB COL7A1	3	20 wk	7-45	**VAS from 6 to 2.5** and an improvement in quality of life evidenced by a more than **30% reduction in DLQI values**
Quintana et al (2024)^6^	DEB COL7A1	1	9 mo	32	Quality of life greatly enhanced, with a **DLQI reduction from 13 to 5.**
Our experience	4 DEB 1 EBS	5	12 mo	1-45	**Mean DLQI reduction of 42.6% observed.** Pruritus values halved at 9 mo and almost completely resolved by 12 mo.
Ichthyosis					
Steuer et al (2020)^9^	NS	1	2-10 mo	32	2 mo: NRS decreased from 9/10 to 2/10. 10 mo: EASI <75%. BSA <50%.
Andreasen et al (2020)^10^	NS	1	1 mo	43	**DLQI decreased from 19 to 2.**
Süßmuth et al (2021)^11^	NS	2	4-12 mo	12	4 mo: NASI decreased from 33 to 11.7. NRS reduced from 8 to 3. Results remained stable after 12 mo of treatment.
				8	**4 mo:** NASI decreased from 50.5 to 18. **NRS reduced from 8 to 3.** Results remained stable after 12 mo of treatment
Our experience	2 LI 1 VI	3	12 mo	7-22	**6 mo:** **DLQI reduction of 40% to 90%.** Reduction in NRS for pain and pruritus by 16.7% to 87.5% and 28.6% to 80%, respectively.

The bold values highlight the main variations observed in the referenced article, which have been further analyzed and discussed in our study.

*BSA*, Body surface area; *COL7A1*, collagen type VII alpha 1 chain; *DLQI*, Dermatology Life Quality Index; *EASI*, Eczema Area and Severity Index; *LI*, lamellar ichthyosis; *NASI*, Netherton Area Severity Index; *NRS*, numerical rating scale; *NS*, Netherton syndrome; *VAS*, visual analog scale; *VI*, vulgar ichthyosis.

### Ichthyosis

Numerous studies have highlighted a correlation between certain recessive ichthyoses, particularly Netherton syndrome (NS), and atopic dermatitis.^1^ These studies also demonstrate the efficacy of dupilumab in managing these conditions.

Specifically, Suẞmuth et al report 2 cases of NS treated with dupilumab, showing a reduction in pruritus NRS from 8 to 2 in 12 months in 1 patient and from 8 to 3 after 10 months in the other.^11^

Similarly, Steuer et al documented a case with a reduction in visual analog scale pruritus from 9 to 2 along with a 75% decrease in disease severity. Andreasen et al reported another case where disease severity decreased from 22.6 to 5.3, alongside an improvement in DLQI from 19 to 2.^9^^,^^10^

Patients with severe recessive forms of ichthyosis often exhibit features resembling NS, such as impaired keratinization, compromised epidermal barrier function, and increased trans-epidermal water loss.

Considering these similarities, we treated our patients with dupilumab, resulting in significant outcomes. DLQI scores improved by 40% to 90%, while NRS scores for pain and pruritus decreased by 16.7% to 87.5% and 28.6% to 80%, respectively, after 6 months of treatment.

A comparative analysis of our findings with existing literature on the efficacy and safety of dupilumab ichthyosis is summarized in Table III.

Notably, no significant adverse events were observed in our study after 12 months of therapy.

In conclusion, these findings highlight dupilumab as a promising therapeutic option for patients with rare genodermatoses, including EB and ichthyoses, beyond NS.

Its modulation of inflammatory cytokines effectively improves clinical signs and symptoms, offering hope for patients with these challenging conditions.

## Conflicts of interest

Dr Caroppo has been a consultant for Leo Pharma, Sanofi Genzyme, AbbVie, Hollister, and Amgen. Dr Belloni Fortina has been a consultant for Almirall, Amgen, Sanofi Genzyme, Pfizer, AbbVie, Leo Pharma, Unifarco, Novartis, and Eli Lilly. Drs Mazzetto, Milan, and Cassalia have no conflicts of interest to declare.
